# When children play, they feel better: organized activity participation and health in adolescents

**DOI:** 10.1186/s12889-015-2427-5

**Published:** 2015-10-24

**Authors:** Petr Badura, Andrea Madarasova Geckova, Dagmar Sigmundova, Jitse P. van Dijk, Sijmen A. Reijneveld

**Affiliations:** Institute of Active Lifestyle, Faculty of Physical Culture, Palacky University Olomouc, Tr. Miru 117, Olomouc, 771 11 Czech Republic; Olomouc Institute for Society and Health, Palacky University, Olomouc, Czech Republic; Department of Health Psychology, Faculty of Medicine, Safarik University, Kosice, Slovakia; Graduate School Kosice Institute for Society and Health, Safarik University, Kosice, Slovakia; Department of Community and Occupational Medicine, University Medical Center Groningen, University of Groningen, Groningen, The Netherlands

**Keywords:** Adolescence, Health, Extracurricular activities, Life satisfaction, Age differences, Gender differences, Sports, Arts #

## Abstract

**Background:**

Participation in organized leisure-time activities (OLTA) has been linked to healthy youth development. This study aimed to assess whether participation in OLTA is associated with both physical and mental health in adolescents, and whether this association differs by pattern of activity participation, age and gender.

**Methods:**

The present study was based on data from the 2013/2014 Health Behaviour in School-aged Children study in the Czech Republic. This data concerned 10,503 adolescents (49.2 % boys) aged 11, 13 and 15. A cluster analysis was carried out to obtain patterns of activity participation and yielded five groups (all-rounders, artists, individual sports, team sports and inactive). The association between participation in types of OLTA and physical and mental health was analysed using logistic regression models adjusted for age and gender. We also assessed interactions between types of OLTA and gender and age.

**Results:**

Participation in OLTA was associated with better self-rated health and higher life satisfaction regardless of gender or age. Participation in team or individual sports was associated with better general health and less frequent health complaints in boys, while participation in art activities was associated with lower occurrence of health complaints in girls and 11-year-olds.

**Conclusion:**

Participation in OLTA is associated with better physical and mental health in adolescents. The association varies by pattern of activity participation and is partly gender- and age-specific.

## Background

Organized leisure-time activities (OLTA) have been identified as a context allowing the individual strengths of adolescents to be aligned with developmental assets [1–3], which stimulates adolescents’ thriving [4–7]. OLTA represent a wide range of activities taking place during leisure time outside the regular school curriculum [8]. They can be characterized as having a structure with defined rules and goals, being supervised by adults, having a regular schedule and putting emphasis on skill-building [5, 6]. Because of these qualities, OLTA are believed to contribute to healthy youth development in contrast to other ways of spending leisure time [9].

The association between health and organized activity participation has been explored as one of the relevant healthy youth development factors in adolescents [8, 10]. Evidence on the relation of OLTA with enhanced physical health is rare [11]. Research has focused mostly on mental health and several studies have documented that it is positively linked to organized activity involvement [12, 13], thus supporting the position of OLTA in healthy youth development.

The effects of activity involvement have been shown to vary by dimensions of OLTA, such as breadth, intensity, duration or engagement [8], as well as by the type of activity [14–16]. The results of some of the studies also suggest age-specific [17, 18] and gender-specific differences [19, 20] in the association between OLTA and adolescent development. However, evidence on such differences regarding health outcomes is lacking, as none of these studies specifically addresses this issue. Thus, OLTA may deserve a place in health promotion in adolescence, but up to now it has not really been clear if the factors of gender and age modify the health outcomes of activity participation. Resnick et al. [21] call for evidence-based support of healthy adolescent development and highlight its benefits for future public health. Therefore, revealing the eventual effects of gender and age on association between OLTA and health of adolescents might provide a useful piece of information from a global health perspective. This study aimed to assess whether participation in OLTA is associated with both physical and mental health in 11-, 13- and 15-year-old adolescents. Further, we aimed to analyse whether these associations differ by a specific pattern of activities, age and gender.

## Methods

### Participants

We obtained data on a nationally representative sample of Czech boys and girls aged 11, 13 and 15 years from the 2013/2014 Health Behaviour in School-Aged Children (HBSC) study. This cross-sectional World Health Organization collaborative study focuses on the health, health behaviours and their socioeconomic determinants among 11-, 13- and 15-year-old children and has been conducted in 4-year intervals since 1983/84. Currently, 44 countries across Europe and North America are members of the HBSC network. More detailed information on the questionnaire used in the last HBSC survey in 2013/2014 can be found in the HBSC International Protocol [22], which can be obtained (upon registration) at the HBSC website: http://www.hbsc.org/methods/.

Schools were selected randomly after stratification by region and type of school (primary schools vs. secondary schools). Out of 244 contacted schools 243 schools agreed to participate (response rate 99.6 %). Then, classes from the 5^th^, 7^th^ and 9^th^ grades, in general corresponding to the age categories of 11-, 13- and 15-year-olds, were selected at random, one from each grade per school.

Data from 14,539 pupils were obtained (response rate 89.2 %). The majority of non-response was due to illness or other reasons, e.g. sports or academic competitions (10.6 %), and 30 children refused to participate in the survey (0.2 %). Because of some unlikely responses, an excess of missing values throughout the questionnaire, or missing information on age, gender or all the responses on OLTA items, 531 questionnaires were excluded. Lastly, according to the HBSC-protocol only adolescents aged 11, 13 and 15 were selected, leading to a final sample of 10,503 respondents.

### Procedure

The data were collected between April and June 2014. The questionnaires were distributed by trained administrators, while the teacher was not present in the classroom to reduce the response bias. Respondents had one school lesson (45 min) dedicated to completing the questionnaire. Participation in the survey was anonymous and voluntary. The data used in this study is not publicly available. Permission to use it was obtained from the Czech HBSC Principal Investigator.

The Czech HBSC study was conducted under auspices of Ministry of Education, Youth and Sports of the Czech Republic and the World Health Organization Country Office in the Czech Republic. The Czech legislation does not require written informed consent for participation in questionnaire surveys. The option to opt out of the study was emphasized to the respondents prior to administration of the questionnaires. The study design was approved by the Ethics Committee of the Faculty of Physical Culture, Palacky University in Olomouc.

### Measures

Life satisfaction as a dimension of mental health was measured by the item ‘*Here is a picture of a ladder. The top of the ladder ‘10’ is the best possible life for you and the bottom ‘0’ is the worst possible life for you. In general, where on the ladder do you feel you stand at the moment?’* [23]. The 11 response categories were dichotomized using a cut-off of 9 to capture the top quartile.

Health complaints were assessed by 4 items (feeling low, irritability or bad temper, feeling nervous, sleeping difficulties) and preceded by the question ‘*In the last 6 months: how often have you had the following…?’* with 5 response categories ranging from *about every day* to *rarely or never*. All four questions were dichotomized using a generally accepted cut-off set to *at least once a week* [24, 25].

Physical health was assessed by the self-rated health measure ‘*Would you say your health is …?’* [26] with 4 response categories *Excellent / Good / Fair / Poor.* Responses were dichotomized using a cut-off point *Excellent* in order to capture the top quartile.

OLTA participation was measured by 6 items dealing with individual types of organized leisure-time activities (team sports, individual sports, art school, youth organizations, recreation/leisure centres, church meeting/singing – including country-specific examples) [27]. The dichotomous question *‘In your free time, do you do any of these organized activities?’* with response categories *yes/no* was followed by the explanatory text: ‘*We mean activities you do in sports or other club or organization’*. Missing answers were considered to represent ‘no’ unless all six OLTA items were missing. Then the respondent was excluded (*n* = 252).

### Statistical analyses

First, we described the composition of the sample and its participation in specific activities and the breadth of the activities. Next, we reduced the amount of possible combinations of particular activity involvement patterns using cluster analysis. Searching for the smallest number of clusters, yet having a reasonable structure, i.e. a value of the average silhouette width exceeding 0.5 [28], we obtained five clusters, which were used in the subsequent analyses. We used chi-square tests to assess the statistical significance of gender and age differences in particular activities, number of concurrent activities and clusters of OLTA and we used one-way ANOVA with a Tukey’s HSD post hoc test to assess the statistical significance of differences in average number of activities with increasing age. Third, we analysed the associations of the binary overall OLTA variable (at least one activity vs. none) and of the clusters of OLTA with the selected health indicators, using logistic regression analyses. We assessed crude associations per health indicator (Model 1). Next, we adjusted for age and gender (Model 2). To assess the moderating effects of gender and age on the associations with health indicators the interactions between gender and OLTA, as well as between age and OLTA, were assessed in the Models 3 and 4. Finally, logistic regressions were run again. All the data were analysed using IBM SPSS 22 for Windows (IBM Corp. Released 2013).

## Results

The characteristics of the sample are presented in Table 1. A vast majority (roughly 4 of 5) of the adolescents was involved in at least one of the six types of organized activities and the average number of activities youth were involved in was 1.52 (*SD* = 1.17). The rate of participation declined with increasing age, regardless of gender, both in terms of the average number (breadth) of activities (*p* < 0.001) and their individual types. Accordingly, the number of inactive adolescents grew as they got older, especially in girls. Over 60 % of adolescents participated in 1 or 2 activities and only about 6 % were engaged in 4 or more activities concurrently. Team sports were the favourite organized activity in boys, while girls preferred artistic pursuits.

**Table 1:** Description of the study population: frequencies of respondents participating in various separate organized leisure-time activities (top section), clusters of activity patterns (middle section), and by number of activities (bottom section) by gender and age

		Gender					Age							Total	
		Boy (*n* = 5170)		Girl (*n* = 5333)			11 (*n* = 3332)		13 (*n* = 3541)		15 (*n* = 3630)			(*n* = 10,503)	
		n	%	n	%	*p*-value	n	%	n	%	n	%	*p*-value	n	%
Participation on each OLTA separately	Team sports	3067	59.3	1960	36.8	<0.001	1709	51.3	1773	50.1	1545	42.6	<0.001	5027	47.9
	Individual sports	1468	28.4	1710	32.1	<0.001	1119	33.6	1088	30.7	971	26.7	<0.001	3178	30.3
	Art school	1012	19.6	2416	45.3	<0.001	1350	40.5	1175	33.2	903	24.9	<0.001	3428	32.6
	Youth org.	733	14.2	640	12.0	<0.001	547	16.4	495	14.0	331	9.1	<0.001	1373	13.1
	Recr./Leisure centre	975	18.9	1050	19.7	ns	838	25.2	727	20.5	460	12.7	<0.001	2025	19.3
	Church	370	7.2	427	8.0	ns	316	9.5	262	7.4	219	6.0	<0.001	797	7.6
OLTA clusters	Active^a^	4238	82.0	4292	80.5	ns	2899	87.0	2960	83.6	2671	73.6	<0.001	8530	81.2
	All-rounders	1578	30.5	1696	31.8	ns	1322	39.7	1143	32.3	809	22.3	<0.001	3274	31.2
	Artists	506	9.8	1447	27.1	<0.001	700	21.0	677	19.1	576	15.9	<0.001	1953	18.6
	Individual sports	738	14.3	634	11.9	<0.001	358	10.7	467	13.2	547	15.1	<0.001	1372	13.1
	Team sports	1416	27.4	515	9.7	<0.001	519	15.6	673	19.0	739	20.4	<0.001	1931	18.4
	Inactive	932	18.0	1041	19.5	ns	433	13.0	581	16.4	959	26.4	<0.001	1973	18.8
Number of activities	1 activity	2142	41.4	1818	34.1	<0.001	1129	33.9	1365	38.5	1466	40.4	<0.001	3960	37.7
	2 activities	1245	24.1	1447	27.1	<0.001	959	28.8	917	25.9	816	22.5	<0.001	2692	25.6
	3 activities	551	10.7	730	13.7	<0.001	519	15.6	483	13.6	279	7.7	<0.001	1281	12.2
	4 activities	198	3.8	207	3.9	ns	205	6.2	127	3.6	73	2.0	<0.001	405	3.9
	5 activities	64	1.2	67	1.3	ns	67	2.0	44	1.2	20	0.6	<0.001	131	1.2
	6 activities	38	0.7	23	0.4	<0.05	20	0.6	24	0.7	17	0.5	ns	61	0.6

% represents the percentage of respondents within the column concerned (gender, age, total) who participated in the given activity or belonged to the particular cluster

*ns* not significant

^a^ the ‘Active’ category in the middle section of the Table represents all the adolescents participating in at least one organized leisure-time activity and is, thus, equal to a sum of the four clusters - All-rounders, Artists, Individual sports and Team sports

The cluster analysis yielded five groups having a reasonable structure. Members of the clusters ‘*inactive*’ and ‘*team sports*’ were inactive or only doing a team sport, respectively. Within the cluster ‘*individual sports*’ approximately half of adolescents participated also in team sports and, similarly, approximately half of the members of the cluster ‘*artists*’ engaged in an individual and/or a team sport in addition to the arts. The cluster ‘*all-rounders*’ comprised all the remaining adolescents and 85 % of its members participated in at least 2 activities.

Table 2 shows odds ratios and 95 % confidence intervals for the associations of the binary participation variables with the health indicators. Being involved in at least one activity was found to be significantly associated with higher life satisfaction, self-rated health and the likelihood of feeling low less than once a week also after adjustment for age and gender (Model 2). The strongest association with participation in OLTA was found for self-rated health resulting in active adolescents being almost twice as likely to perceive their health as excellent. Moreover, they had 1.35 times higher odds of feeling very satisfied. Interactions of participation in OLTA with gender and age were tested next. None of these interactions was statistically significant (not shown).

**Table 2:** Association of binary participation variables with health indicators: odds ratios and 95 % confidence intervals for active vs. inactive adolescents (reference category)

	High life satisfaction (Q1)	Infrequently feeling low (< once a week)	Infrequently irritated/bad tempered (< once a week)	Infrequently feeling nervous (< once a week)	Infrequent sleeping difficulties (< once a week)	Excellent self-rated health (excellent)
Model 1 (univariable)						
≥1 activity vs. inactive	1.51*** (1.35–1.70)	1.33*** (1.17–1.51)	1.12* (1.00–1.25)	1.12* (1.00–1.24)	1.03 (0.91–1.16)	1.94*** (1.70–2.21)
Model 2 (adjusted for age and gender)						
≥1 activity vs. inactive	1.35*** (1.20–1.52)	1.20** (1.06–1.37)	1.08 (0.96–1.20)	1.09 (0.97–1.21)	1.01 (0.90–1.14)	1.93*** (1.69–2.20)

*Q1* top quartile

* *p* < 0.05

** *p* < 0.01

*** *p* < 0.001

Table 3 presents the results of the logistic regressions using the clusters of OLTA as independent variables. The inactive cluster was selected as the reference category for all subsequent analyses. Model 1 shows the crude odd ratios. Except for life satisfaction the interactions with age and/or gender were statistically significant for all other health indicators. Therefore, we do not present results of the age and gender adjusted logistic models regarding the clusters of OLTA without these interactions (Model 2). The members of all four active clusters were more likely to report excellent health compared with the inactive cluster, the strongest association being with the individual sports cluster. Adolescents from all active clusters were also more satisfied with their own lives than the inactive ones. The odds ratios ranged from 1.26 for artists to 1.61 for those in individual sports.

**Table 3:** Associations with health indicators of participation in clusters of organized leisure-time activities compared to inactive: odds ratios and 95 % confidence intervals for the various clusters vs. inactive adolescents

	High life satisfaction	Infrequently feeling low	Infrequently irritated/bad tempered	Infrequently feeling nervous	Infrequent sleeping difficulties	Excellent self-rated health
	(Q1)	(< once a week)	(< once a week)	(< once a week)	(< once a week)	(excellent)
Model 1 (univariable)						
Inactive	1 (reference)	1 (reference)	1 (reference)	1 (reference)	1 (reference)	1 (reference)
All-rounders	1.50 (1.32–1.71)***	1.28 (1.10–1.48)**	1.10 (0.97–1.25)	1.08 (0.96–1.23)	0.96 (0.84–1.09)	1.70 (1.47–1.96)***
Artists	1.39 (1.21–1.61)***	1.05 (0.90–1.24)	1.03 (0.90–1.19)	1.03 (0.90–1.19)	0.94 (0.81–1.09)	1.74 (1.49–2.04)***
Individual sports	1.69 (1.45–1.97)***	1.29 (1.07–1.55)***	1.13 (0.97–1.33)	1.15 (0.99–1.34)	1.01 (0.85–1.19)	2.50 (2.11–2.95)***
Team sports	1.52 (1.32–1.75)***	1.95 (1.63–2.35)***	1.26 (1.09–1.46)**	1.24 (1.08–1.43)**	1.31 (1.12–1.54)**	2.21 (1.89–2.58)***
Model 2 (adjusted for gender and age)						
Inactive	1 (reference)					
All-rounders	1.27 (1.11–1.45)***					
Artists	1.26 (1.08–1.46)**					
Individual sports	1.61 (1.38–1.88)***					
Team sports	1.39 (1.20–1.62)***					
Model 3 (adjusted for gender and age, including interaction with gender)						
Inactive		1 (reference)	1 (reference)	1 (reference)	1 (reference)	1 (reference)
All-rounders		1.19 (0.98–1.43)	1.03 (0.87–1.22)	1.08 (0.91–1.27)	0.96 (0.80–1.15)	1.53 (1.24–1.89)***
Artists		1.21 (1.00–1.48)	1.08 (0.90–1.29)	1.05 (0.88–1.24)	1.09 (0.90–1.31)	1.84 (1.49–2.28)***
Individual sports		1.13 (0.89–1.43)	0.98 (0.79–1.21)	0.98 (0.79–1.21)	0.81 (0.65–1.02)	2.14 (1.67–2.74)***
Team sports		1.16 (0.90–1.50)	0.85 (0.67–1.06)	0.87 (0.70–1.09)	1.05 (0.82–1.34)	1.40 (1.05–1.85)*
Gender M vs. F		2.25 (1.77–2.86)***	1.41 (1.15–1.73)**	1.42 (1.17–1.73)***	1.47 (1.18–1.82)**	1.27 (1.00–1.62)
Artists M vs. F		0.66 (0.45–0.95)*			0.72 (0.52–1.00)*	
Ind. sports M vs. F					1.51 (1.07–2.12)*	
Team sports M vs. F		1.64 (1.12–2.41)*	1.54 (1.14–2.09)**	1.46 (1.09–1.95)*		1.66 (1.18–2.35)**
Model 4 (adjusted for gender and age, including interaction with age)						
Inactive		1 (reference)	1 (reference)	1 (reference)	1 (reference)	1 (reference)
All-rounders		1.17 (0.92–1.49)	1.02 (0.83–1.26)	1.04 (0.84–1.28)	1.03 (0.82–1.28)	1.68 (1.32–2.15)***
Artists		0.92 (0.72–1.18)	0.91 (0.73–1.14)	0.99 (0.79–1.24)	0.91 (0.71–1.15)	1.74 (1.33–2.27)***
Individual sports		1.01 (0.77–1.31)	0.99 (0.78–1.26)	1.05 (0.83–1.33)	1.09 (0.84–1.41)	2.67 (2.07–3.44)***
Team sports		1.31 (1.00–1.70)*	1.03 (0.82–1.28)	1.08 (0.87–1.34)	1.36 (1.06–1.74)*	2.30 (1.81–2.92)***
Age 11- vs. 15-yrs.		1.68 (1.21–2.32)**	1.18 (0.91–1.54)	1.10 (0.85–1.42)	1.16 (0.87–1.53)	0.90 (0.65–1.25)
Age 13 vs. 15-yrs.		1.01 (0.78–1.30)	0.91 (0.73–1.15)	0.87 (0.69–1.09)	1.10 (0.86–1.42)	1.30 (0.99–1.71)
Artists 11- vs. 15-yrs.		1.59 (1.02–2.49)*	1.50 (1.04–2.17)*	1.48 (1.04–2.12)*		
Team sports 13- vs. 1 yrs.						0.68 (0.48-0.98)*

only statistically significant odds ratios for interaction effects of gender and age are presented

*M* males, *F* females, *yrs* years old, *Q1* top quartile

* *p* < 0.05

** *p* < 0.01

*** *p* < 0.001

The interactions of gender with clusters of OLTA (Model 3) regarding its association with health indicators were statistically significant. We found gender differences for three types of health complaints and for self-rated health in team sports and for sleeping difficulties in individual sports. The membership in team sports cluster was found to be more strongly associated with better self-rated health in boys than in girls. Likewise, we observed a stronger association between participation in team and/or individual sports and less frequent health complaints in boys compared with girls. Reversely, girls attending art schools were less likely to face recurrent sleeping difficulties and feel low than boys attending art schools.

Model 4 assessed the interaction effects of age with cluster of OLTA. A stronger association between participation in art pursuits and a lower occurrence of health complaints (feeling low, irritability/bad temper and feeling nervous) was found in 11-year-olds compared with 15-year-olds. Furthermore, the relationship between self-rated health and involvement in team sports was stronger for the oldest age category of adolescents than for those aged 13. None of the other interactions between age and clusters of activity patterns was statistically significant. As mentioned above, the interaction effects of age and gender were not statistically significant for life satisfaction; therefore the results for this indicator are not presented in Models 3 and 4.

## Discussion

Participation in OLTA was associated with enhanced physical and mental health among all adolescents independently of the type of OLTA. The associations between particular clusters of OLTA and less frequent occurrence of specific health complaints varied by gender and age.

As expected, we found that being engaged in one or more OLTA, regardless of their type, age or gender, was associated with higher life satisfaction and better self-rated health. This is in line with the evidence that states that in general any level of involvement is better than no involvement [29, 30]. However, the associations between particular clusters of OLTA and lower prevalence of health complaints were observed to differ by gender and age. This indicates that the actual type of activity matters, as previously pinpointed by Agans and Geldhof [31].

Unlike other studies [32, 33] we observed the strongest associations with healthy development indicators in adolescents engaged solely in sports and not in those having different patterns of activity involvement, i.e.’ all-rounders’ and ‘artists’ clusters. This contradicts the broadly recognized premise that the more contexts children are involved in, the more developmental opportunities they have [13, 34–36]. Physical activity may be a significant element underlying this finding as it offers undisputable benefits for physical and mental health [37–39]. Those participating only in sports perhaps engage in physical activities more frequently and intensely than those who are involved in different types of OLTA, thus, enhancing their health-promoting effects. The link of other leisure pursuits and health in adolescents appears to be weaker, which accords with the results of Zambon et al. [11].

Next, we found participation in individual and team sports to be associated with fewer health complaints only in boys. Physical activity has been documented to reduce symptoms of nervousness, irritability and sadness more in females than in males [40]. Therefore, we believe the weaker association between organized sports and health complaints in adolescent girls might be attributable mostly to motivational and social factors. First, compared with boys European girls do not consider competition/achievement in physical activity to be as important [41, 42]. This is actually in conflict with primary goal of sports (to win) as a performance- and success-centred setting. Second, boys consider conflict, which often arises in a competitive environment, to be a more natural part of their sport friendship than girls, who rather appreciate companionship, intimacy or pleasant play [43]. These discrepancies might lead to higher irritability or nervousness in girls, which could explain the relatively stronger association with a lower frequency of these health complaints in boys. Based on our results, it may be useful, especially in girls, to treat individual and team sports as separate contexts of healthy youth development, with each having specific advantages as has already been suggested by Hansen et al. [14].

Regarding the interaction effect of age, the most prominent differences in the associations between participation in OLTA and the occurrence of health complaints were observed in the artist cluster. The associations with less frequently feeling low, nervousness and irritability were found in 11-year-old artists but not in those aged 15. In their systematic review, Bungay & Vella-Burrows [44] reported that interventions using creative activities were supportive of well-being in youth without mentioning any age-specific data. Nevertheless, art performers have in general been shown to be more prone to irritability, anxiety [45], neuroticism [46] and mood disorders [47]. Since we observed a noteworthy decline of participation in art activities with increasing age, it is possible that only the “genuine” artists, who are more apt to suffer from the above-mentioned symptoms, maintain their involvement. This could subsequently result in the diminishing of the associations for the oldest age category.

### Strengths and limitations

The present study has several strengths. The most important are its sample size and the representativeness of the adolescent sample. Moreover, it uses the well-established HBSC methodology that is being developed by expert groups on a continuous basis. To the best of our knowledge it is also the first study in Europe using such a systematic classification of OLTA and dealing with age and gender differences in more detail.

Our findings also need to be interpreted in light of some limitations. First, we did not explore other dimensions of OLTA, such as frequency, duration, intensity and quality. These have been shown to affect the developmental outcomes [8]; therefore the associations found in the present study might have been partly attributable to some of these characteristics. Second, the cross-sectional design does not allow us to draw any conclusions on causal relationships. It might be that being healthy and satisfied is a prerequisite for entering an organized activity and not its consequence [48]. Third, all the analyses were based on self-reported data, which is more susceptible to recall bias, though measures have in general been well-validated [22]. Fourth, we found sports participation to be strongly associated with health but we could not adjust for physical activity. We could thus not determine to which degree this association was due to physical activity in itself, or due to the organisational aspect of sports participation.

### Implications

In general, the results of our study support the contribution of OLTA to adolescents’ health. However, the gender- and age-related differences in particular patterns of activities suggest that sports participation may reduce the prevalence of health complaints primarily in boys. On the other hand, girls and younger adolescents seem to benefit more from art activities.

Future research should concentrate on differences in relationship between particular types of OLTA and relevant developmental indicators. It should be also focused on unveiling the causal pathways between participation in organized-leisure time activities and health.

## Conclusions

Organized activities done in leisure time are associated with better physical and mental health among adolescents, and this association is partly gender- and age-specific. While boys and older kids might benefit more from participating in team or individual sports, participation in art schools was associated with better health outcomes in girls and younger kids.

## Abbreviations

ANOVA: Analysis of variance
HBSC: Health Behaviour in School-Aged Children
OLTA: Organized leisure-time activities
SPSS: Statistical Package for the Social Sciences
Tukey’s HSD test: Tukey’s honest significance difference test
